# Atypical changes in *Candida albicans* cells treated with the Venetin-1 complex from earthworm coelomic fluid

**DOI:** 10.1038/s41598-023-29728-0

**Published:** 2023-02-17

**Authors:** Sylwia Wójcik-Mieszawska, Kinga Lewtak, Weronika Sofińska-Chmiel, Jerzy Wydrych, Marta J. Fiołka

**Affiliations:** 1 Department of Immunobiology, Institute of Biological Sciences, Faculty of Biology and Biotechnology, Maria Curie-Skłodowska University, Lublin, Poland; 2 Department of Cell Biology, Institute of Biological Sciences, Faculty of Biology and Biotechnology, Maria Curie-Skłodowska University, Lublin, Poland; 3 Analytical Laboratory, Institute of Chemical Sciences, Faculty of Chemistry, Maria Curie-Skłodowska University, Lublin, Poland; 4 Department of Functional Anatomy and Cytobiology, Institute of Biological Sciences, Faculty of Biology and Biotechnology, Maria Curie-Skłodowska University, Lublin, Poland

**Keywords:** Biotechnology, Cell biology, Drug discovery, Microbiology, Diseases

## Abstract

In the present research, the effect of a protein-polysaccharide complex Venetin-1 obtained from the coelomic fluid of *Dendrobaena veneta* earthworm on *Candida albicans* cells was characterized. The compound destroyed fungal cells without showing cytotoxicity to human skin fibroblasts, which was demonstrated in earlier studies. Since it had an effect on the fungal cell wall and membrane, this complex was compared with the known antifungal antibiotic fluconazole. Both preparations disturbed the division of yeast cells and resulted in the formation of aggregates and chains of unseparated cells, which was illustrated by staining with fluorochromes. Fluorescent staining of the cell wall with Calcofluor white facilitated comparison of the types of aggregates formed after the action of both substances. The analysis performed with the use of Congo red showed that Venetin-1 exposed deeper layers of the cell wall, whereas no such effect was visible after the use of fluconazole. The FTIR analysis confirmed changes in the mannoprotein layer of the cell wall after the application of the Venetin-1 complex. Staining with Rhodamine 123 and the use of flow cytometry allowed comparison of changes in the mitochondria. Significantly elongated mitochondria were observed after the Venetin-1 application, but not after the application of the classic antibiotic. Phase contrast microscopy revealed vacuole enlargement after the Venetin-1 application. The flow cytometry analysis of *C. albicans* cells treated with Venetin-1 and fluconazole showed that both substances caused a significant decrease in cell viability.

## Introduction

Medical advances have accelerated considerably in recent years both in terms of surgery and the treatment of chronic diseases and infections. Thus, the number of patients with weakened immunity as a result of surgical treatment, antibiotic therapy, chemotherapy, or treatment in hospital intensive care units (ICU) has increased^1–4^. The dark side of this situation is the increasing number of nosocomial infections. They are caused by, inter alia, the overuse of antibiotics, which leads to the development of microbial resistance to commonly used drugs^5^. Infections with nosocomial pathogens extend the patient's stay in a medical facility and increase the costs of treatment^2,3,6^.

*Candida* fungi, especially *Candida albicans*, are an example of pathogens causing such infections. *C. albicans* is an opportunistic pathogen living commensally on the surface of skin and mucous membranes and as part of the intestinal microflora in healthy subjects^7–9^. In immunocompromised patients, this fungus causes infections of the skin and mucous membranes as well as life-threatening systemic candidiasis and systemic infections^7,10,11^. This yeast is the fourth most common ICU pathogen in the US and the first one in Europe^3,4^. *Candida* blood infections associated with ICU admissions account for approximately 40% of cases, with 9.3% of patients developing sepsis^2^.

*C. albicans* owes its success as a pathogen to the high plasticity of the genome, the ability to create biofilms, the production of lytic enzymes, and the ability to adhere to surfaces^7,9,11–13^. Biofilms are particularly dangerous to patients. They can be formed on both natural and artificial surfaces. Biofilms are resistant to high concentrations of antifungal antibiotics, which makes them difficult to remove, and they are a source of pathogenic cells that can create new infection foci in the body^7,14,15^. It is estimated that the mortality rate in subjects infected with *Candida* may be as high as 40%^2,3,16,17^.

Fluconazole is one of the most commonly used antibiotics in the treatment of candidiasis. It is an azole antibiotic which acts by inhibiting the enzyme 14-α-sterol demethylase involved in the synthesis of membrane sterols^7^. This results in a change in the properties and structure of the cell membrane, which leads to inhibition of the growth of the fungus. Despite the low toxicity of fluconazole to host cells, the drug is increasingly being reported as ineffective in the treatment of candidiasis^7^. This is caused by the development of antibiotic resistance by microorganisms through mutations in their genetic material leading to an increase in the number of multi-drug pumps on the surface of their cells^7,18^.

Due to the tendency of *C. albicans* to develop antibiotic resistance, it is necessary to search for alternative preparations that can be used in the treatment of candidiasis. The natural environment, which has inspired natural medicine for hundreds of years, can be used as a source of such substances. Earthworms are organisms that have long been used to treat fungal infections in traditional Far Eastern medicine. They have also been used to treat many other diseases, such as jaundice or cardiovascular diseases, and to increase immunity^19,20^. Pastes, powders, and extracts prepared from earthworms and their symbionts have documented activity against *Candida* yeasts and bacteria^20–31^. Earthworm coelomic fluid, fractions separated from the fluid, and extracts from these invertebrates show anticoagulant, anti-inflammatory, and antitumor activity as well^19,32,33^.

Our studies showed that the protein-polysaccharide complex described previously as a fraction from the coelomic fluid had no cytotoxic activity against normal human fibroblasts^34^. The Venetin-1 complex showed antitumor activity against non-small lung cancer^35^ and colon cancer^36^ and exerted a multi-pathway antiplatelet effect without coagulopathy and cytotoxicity^37^. The active compound was separated from the earthworm coelomic fluid after elimination of its cytotoxicity using incubation at elevated temperature^33^. After this process, the antitumor and antifungal activity was maintained, and normal cells were spared at the same time. In terms of chemistry, the preparation had the same composition at each analyzed point, and was chemically characterized in previous publications as a protein-polysaccharide compound and a microparticle with dimensions of 58.23 ± 3.93 nm^34,35^.

Since the obtained complex acts on the cell wall and membrane of *C. albicans*, as shown in earlier publications, it seemed advisable to analyze this effect in comparison to the standard antibiotic, which also interacts with the fungal cell membrane. Given the increasing antibiotic resistance of microorganisms, the results of studies on the antifungal activity of the complex suggest that the complex from coelomic fluid may be a valuable preparation in the fight against diseases caused by fungi of the genus *Candida*.

## Materials and methods

### Earthworms breeding

*Dendrobaena venet*a earthworms were used in this research. These invertebrates were reared in supervised conditions in the Immunobiology Department of Maria Curie-Skłodowska University in Lublin (Poland). The annelids were kept in containers with a capacity of 3L. The containers were filled up with compost soil and kept in the dark with air flow at 25 °C and 70–80% humidity. The earthworms were fed three times a week with green tea leaves, cellulose, and boiled vegetables. Only mature earthworms were selected for the experiments.

### Isolation of Venetin-1 from earthworm coelomic fluid

Water-rinsed earthworms were kept on moist lignin for 24 h. The coelomic fluid (CF) was obtained from groups of 10 individuals immersed in a small amount of 0.9% NaCl in order to maintain electrical conductivity. The fluid was obtained after electrostimulation with a current of 4.5 V applied for 1 min. The obtained CF was centrifuged (6000×*g*, 10 min) in order to separate coelomocytes. The supernatant was then filtered through 0.22 Millipore filters and heated at 70 °C for 10 min. The cell-free CF was then transferred into a dialysis bag with a 12–14 kDa cut-off and dialyzed overnight at 4 °C against distilled water. The sterile complex was transferred into Eppendorf tubes, lyophilized, and stored at -20 °C. The protein concentration in the Venetin-1 complex was determined with the Bradford method^38^.

### Preparation of *C. albicans *cell culture with Venetin-1 and fluconazole

The clinical isolate of the *C. albicans* wild type strain used for the microbiological analysis was gifted by Prof. A. Kędzia from Department of Oral Microbiology, Medical University of Gdańsk. Before the experiments, the fungal culture was pre-grown in liquid Sabouraud medium at 28 °C. After 24 h of incubation, 30 µL of the *C. albicans* culture (10^7^ CFU in the logarithmic growth phase) was added into 150 µL of YPD poor medium supplemented with streptomycin sulphate (Sigma) at a final concentration of 0.17 mg mL^−1^ and Venetin-1 or fluconazole. The final concentrations of Venetin-1 and fluconazole used in the experiments were 25, 50, and 100 µg mL^−1^ and 5, 10, and 20 µg mL^−1^, respectively. The samples were incubated at 37 °C for 48 h with shaking.

### Microscopic analysis of *C. albicans* cells after Venetin-1 and fluconazole treatment

Cell cultures intended for the microscopy analysis were prepared as described in “Preparation of*C. albicans*cell culture with Venetin-1 and fluconazole” section of the Materials and methods. Phase contrast light microscopy analyses were conducted using unstained *C. albicans* cells. Four fluorochromes: Congo red, Calcofluor white, Hoechst 33342 with Propidium iodide mixture, and Rhodamine 123 were used for fluorescence microscopy. The fluorescence microscopy analysis and the phase contrast analysis were conducted with the use of a confocal laser scanning microscope (Carl Zeiss, Jena, Germany). All excitation wavelengths used in the analysis and visualized structures are presented in Table 1.

**Table 1:** Fluorescent dyes and their properties.

Fluorochrome	Excitation wavelength [nm]	Dyed structure	Fluorescence color
Congo red	543	Cell wall β-1,4 glucans	Red
Calcofluor white	260	Cell wall chitin	Blue
Hoechst 33342 + Propidium iodide	488	Genetic material in the nucleus	Blue (regular cells) White (apoptotic cells) Red (necrotic cells)
Rhodamine 123	507	Membrane of active mitochondria	Green

#### Calcofluor white staining

Calcofluor white (Fluka) binds to chitin in the fungal cell wall. The fluorochrome (20% aqueous solution) was incubated with a 1:1 suspension of the analyzed cells for 10 min at room temperature in the dark. Then, 2 µL of the suspension was transferred onto a microscope slide and observed. Aggregates present in all cultures were counted after analysis of 1000 forms. The experiment was repeated three times. The forms identified were single cells, doublets, triplets, quadruplets, chains, small aggregates (< 10 cells), large aggregates (> 10 cells), and hyphae/pseudohyphae.

#### Congo red staining

Congo red dye (Sigma-Aldrich) was used to visualize β-1,4 glucans 1,- of the cell wall. Aqueous solution (2%) of the dye was mixed with the suspension of analyzed *C. albicans* cells in a 1:1 ratio. After 3 min incubation in the dark at room temperature, 2 µL of the preparation was transferred onto a slide and observed.

#### Hoechst 33342 and Propidium iodide staining

A Hoechst 33342 (Sigma-Aldrich) and Propidium iodide (Sigma-Aldrich) staining mixture was prepared and added to the fungal cultures in 1:1 ratio and then incubated at 37 °C for 5 min in the dark. 2 µL of the cell suspension was analyzed under the microscope. The dyes were used for visualization of genetic material of the cells in their current state: regular cells showed blue fluorescence of nuclei, apoptotic cells exhibited bright white florescence of fragmented genetic material, and necrotic cells had red fluorescence^39,40^.

#### Rhodamine 123 staining

The Rhodamine 123 (ThermoFisher Scientific) dye is used to stain membranes of properly functioning mitochondria. Active mitochondria exhibit green fluorescence. Cells with disturbed functioning of mitochondria do not have membrane potential and do not emit fluorescence. Rhodamine 123 was used in two concentrations: 120 µg mL^−1^ for the control cells and cultures incubated with Venetin-1 and 30 µg mL^−1^ for cells treated with fluconazole. The use of the lower dose of the dye for fluconazole was related to the increased permeability of the *C. albicans* cell wall after antibiotic treatment. The negative control was performed using sodium azide, which is an inhibitor of the mitochondrial respiration pathway^41^. A 1.3% sodium azide (Sigma) solution was incubated with the control cells in a ratio of 1:1 for 10 min at room temperature. All fungal cells, i.e. the negative and positive control and cells treated with fluconazole and Venetin-1, were incubated with Rhodamine 123 at different concentrations at 37 °C for 20 min and then washed 3 times with sterile water. The samples were transferred onto microscopic slides and observed.

### Cryo-SEM analysis

The control culture cells and cells treated with Venetin-1 at the concentration of 100 µg mL^−1^ were centrifuged (room temperature, 6000×*g*, 10 min) and the supernatant was discarded. The pellet was suspended in 200 µL of a GH solution (composed of glucose, HEPES, and sterile water) and centrifuged again. Then, the supernatant was removed and 20 µL of the GH solution was added. Next, the cells were transferred into a sublimation chamber with a temperature of − 92 °C for 12 min. After that, the frozen cultures were cut in the preparation chamber and analyzed with a ZEISS Ultra Plus Field Emission Microscope (Carl Zeiss, Germany) with electron high tension (EHT) 5 kV and under magnification 30.000 × ^39,42^.

### SEM analysis

Cell cultures prepared as described in “Preparation of*C. albicans*cell culture with Venetin-1 and fluconazole” section of Materials and methods were centrifuged at room temperature at 2500×*g* for 10 min. The supernatant was discarded and the pellet was suspended in 70 µL of a fixing solution (10 ml phosphate buffer pH = 7, 10 ml glutaraldehyde 10%, 200 mg saccharose) and incubated for 2 h at room temperature. Then, the fixing solution was discarded and 0.1 M phosphate buffer pH = 7 was added. After centrifugation (2500×*g*, 30 min, room temperature), a 1.5% OsO_4_ solution was added and incubated with the cells for 30 min at room temperature. After that, the fungal cells were centrifuged and the supernatant was removed. Then, the cultures were washed with phosphate buffer and centrifuged. The cells prepared in this way were dehydrated in a series of acetone dilutions (15%, 30%, 50%, 70%, 100%), transferred onto SEM stages with carbon discs, and dried in a desiccator for 24 h in the presence of roasted silicone gel (for 2 h at 180 °C). Afterwards, the stages with the probes were coated with a gold layer using a K550X sputtering machine (Quorum Technologies, United Kingdom) and observed with the use of a scanning electron microscope Tescan Vega 3 (Tescan Orsay Holding, Czech Republic)^39^.

### Flow cytometry analysis

The flow cytometry analysis used in our research allowed discrimination between living, apoptotic, and dead cells. The cell cultures were prepared for the analysis as described in “Preparation of*C. albicans*cell culture with Venetin-1 and fluconazole” section of Materials and methods. Next, 20 µL of the cell culture suspension was added to 780 µL of ViaCount reagent (Luminex, USA) and incubated for 5 min in the dark at room temperature. The emission recording channels used for ViaCount were red and yellow. 5000 cells were counted for each sample. The results were analyzed with GuavaSoft software with the Via count designation and viability counted with the EasyFit tool, which allows distinguishing viable and apoptotic cells overlapping each other. The flow cytometry analysis was performed using a Guava easyCyte flow cytometer (Luminex, USA).

The flow cytometry analysis of active mitochondria in *C. albicans* cells was performed with the use of Rhodamine 123 (water solution 20 µg mL^−1^). The cell suspension prepared as described in “Preparation of*C. albicans*cell culture with Venetin-1 and fluconazole” section of Materials and methods was mixed with a fluorochrome solution and incubated at room temperature in the dark for 10 min^41^. The channels used for emission recording were Forward Scatter and Green. The results were analyzed using the InCyte program. Statistical significance analysis were performed using Statistica program and HSD Tuckey test.

### FTIR spectroscopy analysis

FTIR spectroscopy is one of the most frequently used research techniques allowing the observation of the chemical structure of organic and inorganic compounds. It is also widely used in studies of biological systems. The main advantage of the FTIR technique compared to other spectroscopic methods is the fact that almost all chemical compounds exhibit characteristic absorption of radiation in the IR spectral region; hence, they can be analyzed both qualitatively and quantitatively^43.^ Infrared spectroscopy examines the absorption of radiation associated with the excitation of the vibrational levels of molecules, which facilitates observation of changes in the area of chemical bonds. In order to observe the changes taking place in the *C. albicans* cells after the treatment with Venetin-1 at a concentration of 100 µg mL^−1^ and fluconazole at a concentration of 20 µg mL^−1^, FTIR spectra of the prepared samples were obtained. The tests were carried out using the ATR technique directly from the sample surface at room temperature. The spectrometer was recorded using a Thermo Nicolet 8700 FTIR spectrometer with a Smart Orbit™ diamond ATR attachment equipped with a DTGS (Deuterated Triglycine Sulphate) detector. The spectra were recorded in the mid-infrared range: 4000–650 cm^−1^ with a spectral resolution of 4 cm^−1^. The spectra obtained were subjected to baseline correction and scaled normalization.

## Results

### *C. albicans* cell aggregates identified by Calcofluor white staining

**Figure 1:**
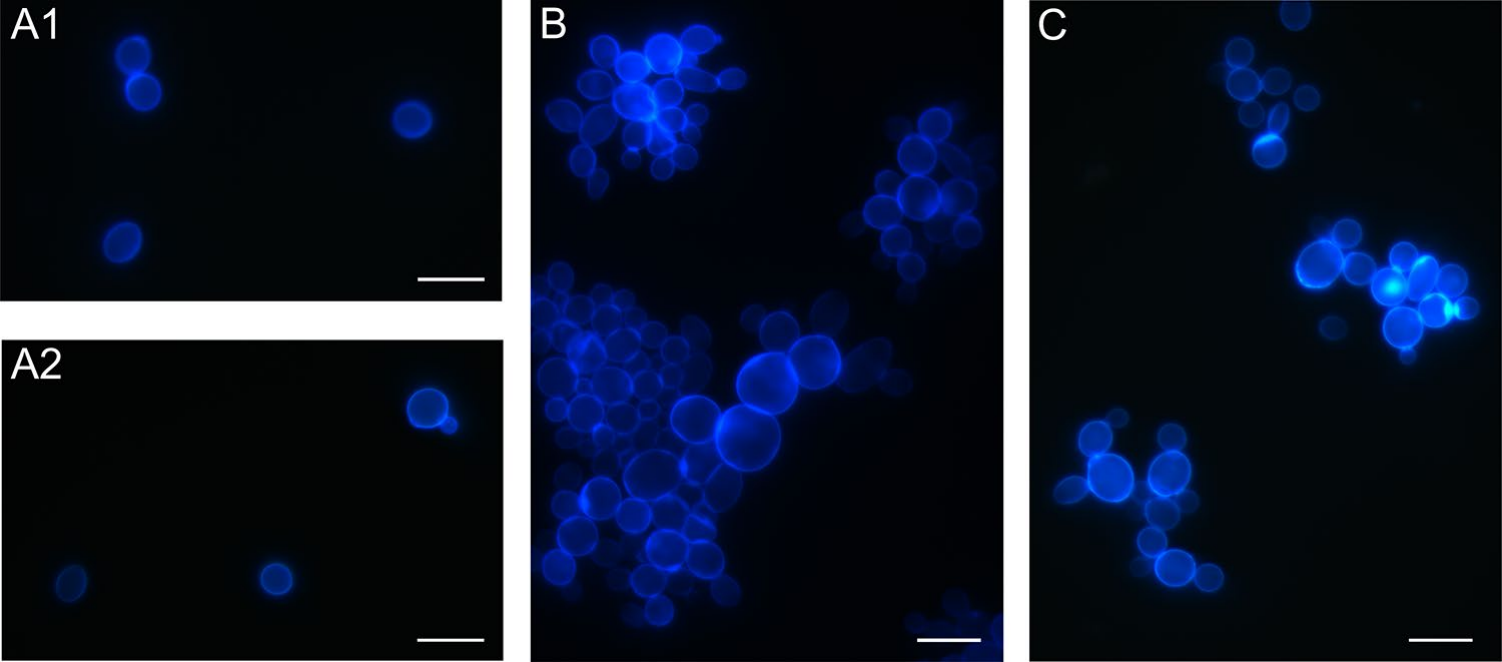
Images of aggregates of *C. albicans* cells after the staining with Calcofluor white: (**A1**)–(**A2**)-control *C. albicans* cells; (**B**)- *C. albicans* cells after treatment with Venetin-1 at 100 µg mL^−1^; (**C**)- *C. albicans* cells after incubation with fluconazole at 20 µg mL^-1^. The scale bar represents 5 µm.

**Figure 2:**
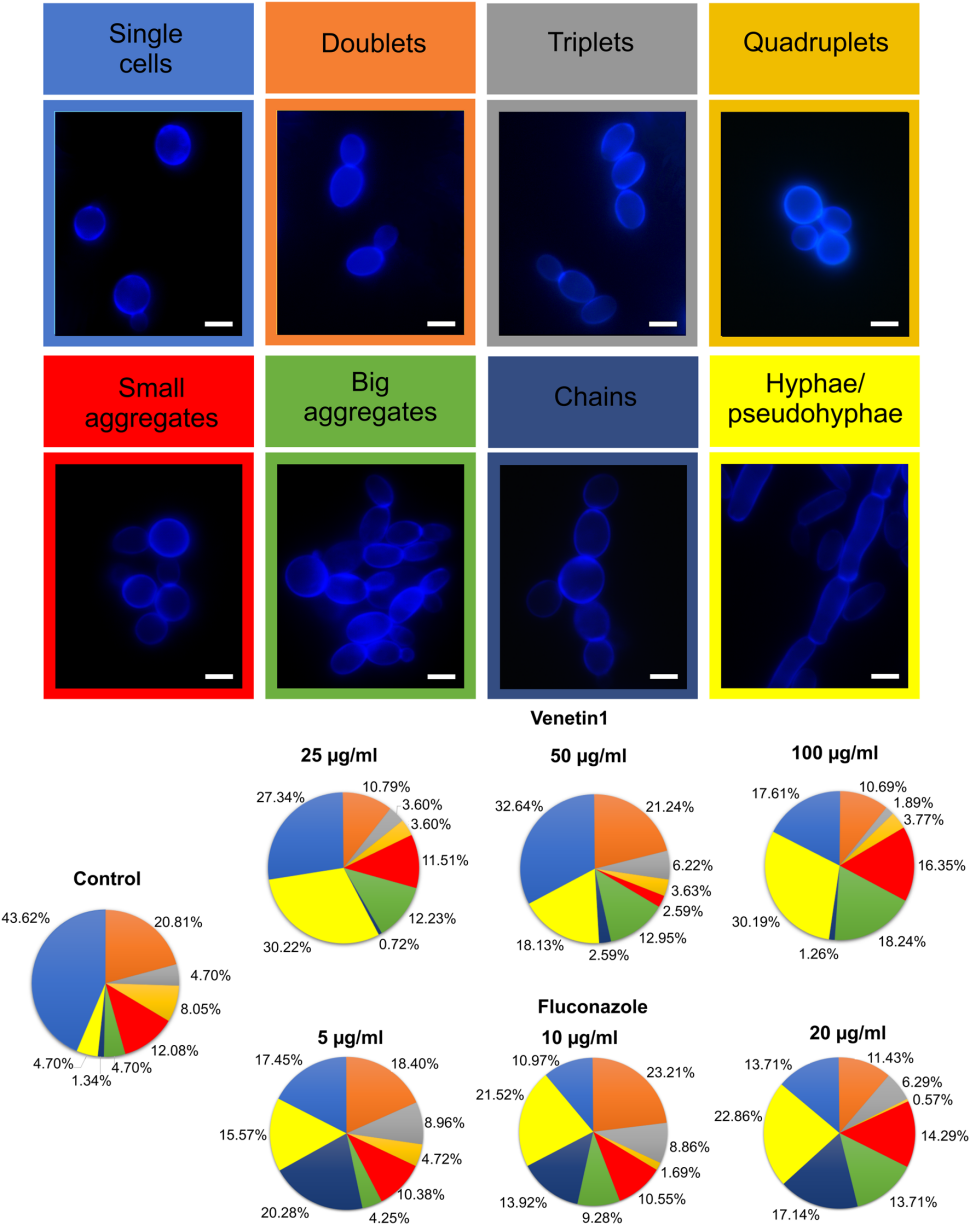
Types of aggregates in the control *C. albicans* culture and cultures incubated with different concentrations of Venetin-1 and fluconazole. The colors presented on the upper panel correspond to the colors on the pie charts. The scale bar represents 2 µm.

The Calcofluor white staining resulting in intense luminosity of the cell wall distinguished different aggregates in the culture of *C. albicans* cells (Fig. 1). *C. albicans* cells were incubated with Venetin-1 and fluconazole at different concentrations; afterwards, the individual aggregates were counted and their percentage is shown in Fig. 2. The number of single cells decreased from 43.72% in the control culture to 17.61% in the culture incubated with Venetin-1 at a concentration of 100 µg mL^−1^ and to 10.79% in the culture treated with fluconazole at a concentration of 10 µg mL^−1^. Twice as many fungal triplets were observed in the culture incubated with fluconazole (at a concentration of 5 µg mL^−1^), compared to the control culture, i.e. 8.96% and 4.70%, respectively. There were also fewer quadruplets formed in the cultures incubated with both Venetin-1 and fluconazole. The percentage of small aggregates (composed of less than 10 cells) did not differ between the control culture and the cultures treated with Venetin-1 and fluconazole. A significant rise was observed in the formation of big aggregates (comprising 10 cells and more) from 4.7% in the control culture to 18.24% in the culture incubated with Venetin-1 at a concentration of 100 µg mL^−1^. The percentage of chains formed in the cell culture incubated with fluconazole (20 µg mL^−1^) also changed (from 1.34 to 17.14%) in comparison to the control culture. A significant increase in the number of hyphae/pseudohyphae was also observed after the incubation with both preparations, reaching 4.7% in the control culture, 30.19% in the culture incubated with Venetin-1 (at 100 µg mL^−1^) and 22.86% after the incubation with fluconazole (at 20 µg mL^−1^) (Fig. 1).

### Visualization of the *C. albicans* cell wall after staining with Congo red

**Figure 3:**
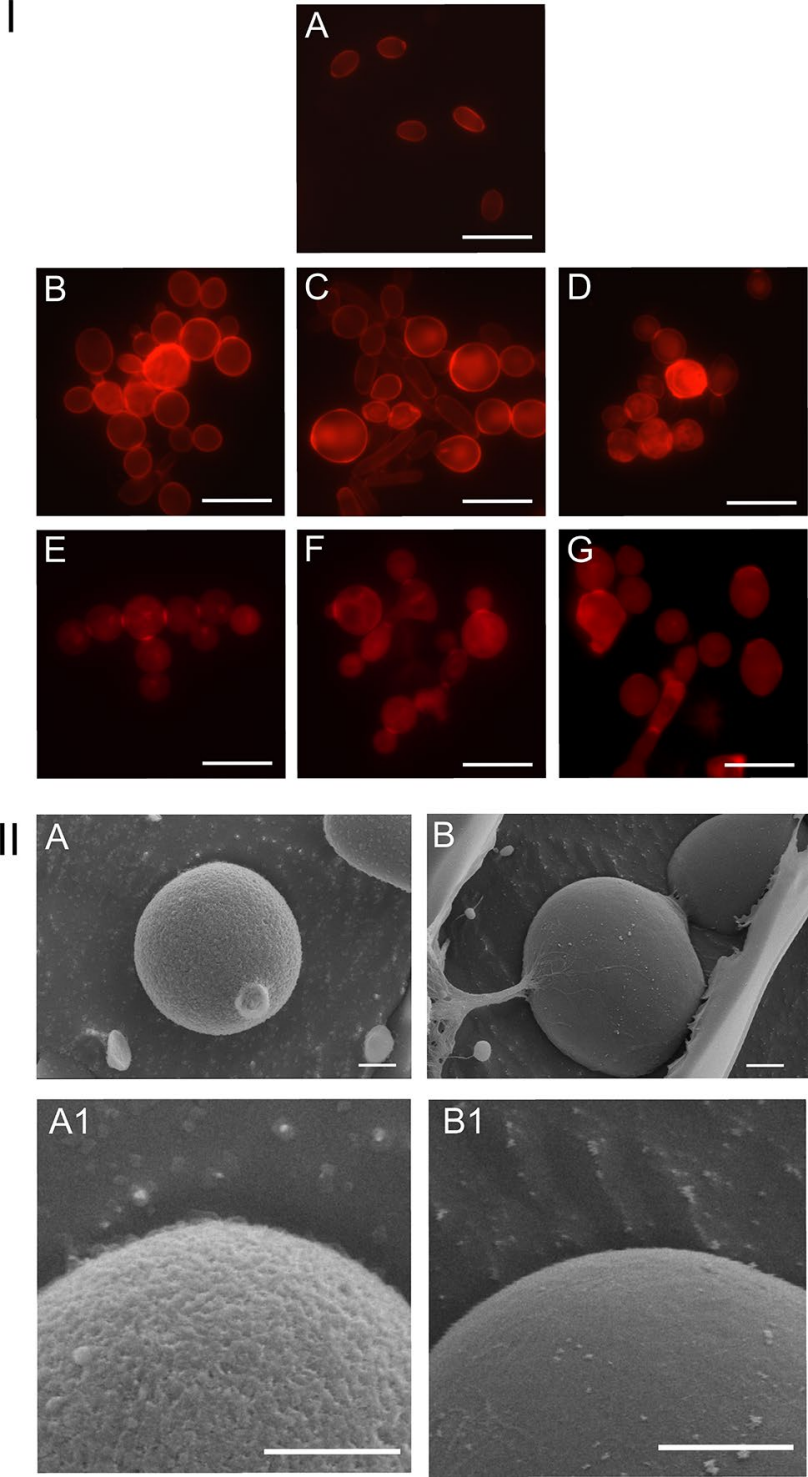
I. *C. albicans* cells after the incubation with Venetin-1 and fluconazole, stained with Congo red dye: (**A**)—cells of the control culture; (**B**)—cells after the incubation with Venetin-1 at 25 µg mL^−1^; (**C**)—with Venetin-1 at 50 µg mL^−1^; (**D**)—with Venetin-1 at 100 µg mL^−1^; (**E**)—cells after the incubation with fluconazole at 5 µg mL^−1^; (**F**)—with fluconazole at 10 µg mL^−1^; (**G**)—with fluconazole at 20 µg mL^−1^. The scale bar represents 10 µm. II. Cryo-SEM images of *C. albicans* cells; A–A1—cells of the control culture, B–B1—cells after the incubation with Venetin-1 at 100 µg mL^-1^. The scale bar represents 1 µm.

The Congo red staining of the control showed that the cells were round or oval with slight red fluorescence of the cell wall (Fig. 3 I A). The cells observed after the incubation with Venetin-1 at a concentration of 25 µg mL^-1^ were larger, with stronger fluorescence (Fig. 3 I B). Picture I C shows changes in the cell shape as well as an increase in their size after the treatment with Venetin-1 at 50 µg mL^−1^ (Fig. 3 I C). Non-separated cells and red fluorescence were observed on the whole cell surface after the incubation with Venetin-1 at 100 µg mL^−1^ (Fig. 3 I D). The cultures subjected to the treatment with fluconazole at concentrations of 5 and 10 µg mL^−1^ exhibited changes in the shape and size of the cells and branched chains of non-separated cells with brighter fluorescence than in the control cultures (Fig. 3 I E- F). Cultures incubated with fluconazole at 20 µg mL^−1^ had additionally fluorescent connections between the chain-forming cells (Fig. 3 I G). Moreover, pseudohyphae and cells with bud scars were visible in this sample. The incubation with all the fluconazole concentrations did not result in stronger fluorescence, compared to that observed after the use of Venetin-1.

The Cryo-SEM technique was used to visualize the cell wall surface. The images in Fig. 3 II A, A1 show the surface of the *C. albicans* cell wall in the control culture. The surface was clearly lumpy. The use of Venetin-1 at the highest concentration (100 µg mL^−1^) had a wall surface smoothing effect, which may indicate a disturbance in the structure of the fungus wall (Fig. 3 II B, B1).

### Analysis of *C. albicans* cells using Hoechst 33342 and Propidium iodide

The use of the mixture of Hoechst 33342 and Propidium iodide facilitated discrimination between normal, apoptotic, and necrotic cells. Normal cell nuclei showed blue fluorescence and had a regular shape. Apoptotic cells had fragmented genetic material with bright white-blue fluorescence, while necrotic cells were characterized by the red color of the nuclear material.

**Figure 4:**
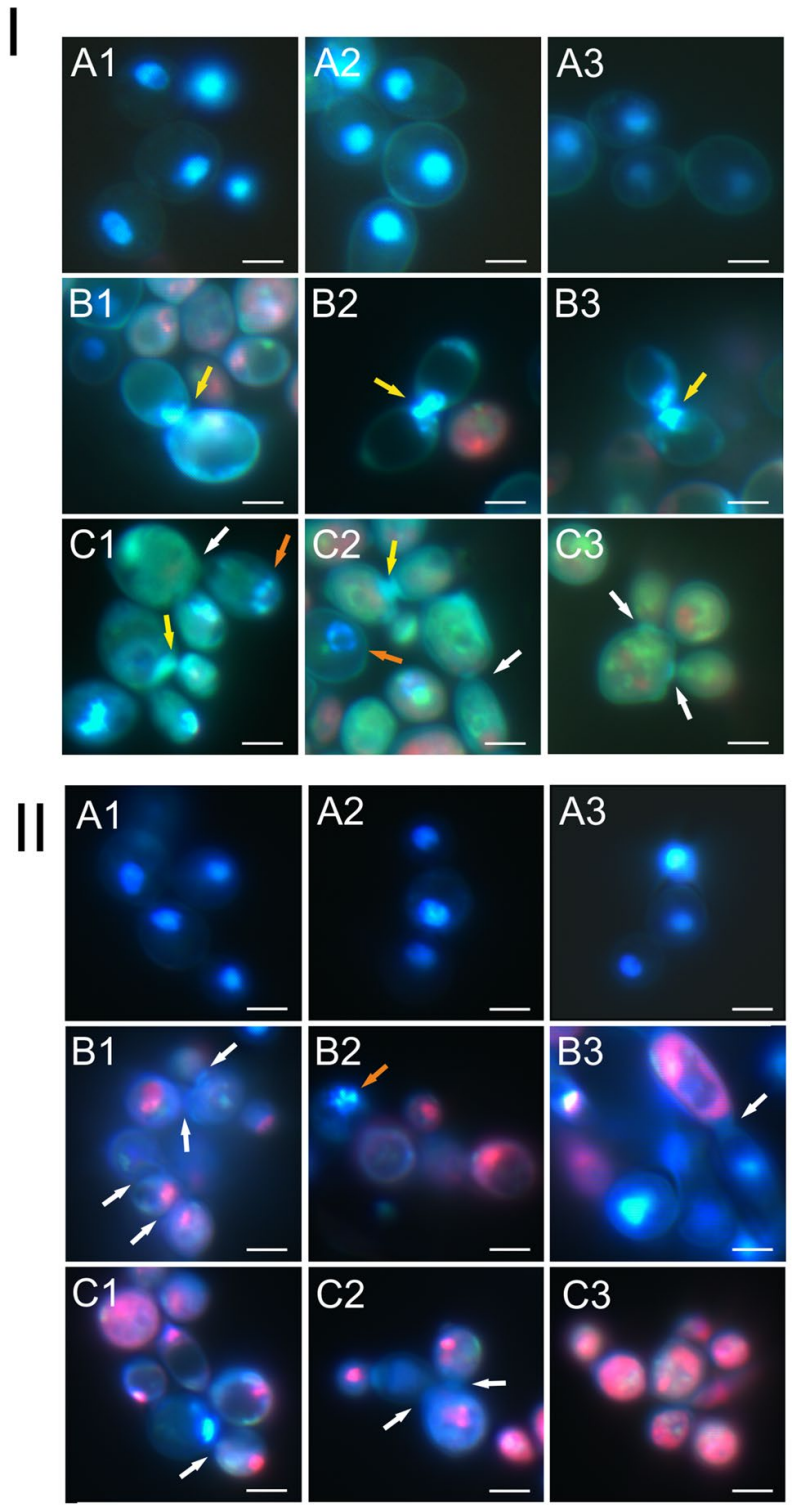
*C. albicans* cells after the incubation with Venetin-1 and fluconazole stained with a mixture of Hoechst 33342 and Propidium iodide dyes; I- A1–A3—control culture cells; B1–B3—cells after the incubation with Venetin-1 at 50 µg mL^−1^; C1–C3—with Venetin-1 at 100 µg mL^-1^; II- A1–A3—control culture cells, B1–B3—cells after the incubation with fluconazole at 10 µg mL^−1^, C1–C3—with fluconazole at 20 µg mL^-1^. The yellow arrows indicate non-separated genetic material of the cells after cell division; the white arrows indicate incomplete separation of cells after division; orange arrows show apoptotic cells. The scale bar represents 5 µm.

The control *C. albicans* cells had oval nuclei with blue fluorescence (Fig. 4 I A1–A3 and II A1–A3). The cells treated with Venetin-1 at the concentration of 50 µg mL^−1^ had genetic material distributed over the junction of two cells, i.e. parent and daughter cells, suggesting disturbances in the cell division process (Fig. 4 I B1–B3, marked with yellow arrows). The cells after the incubation with Venetin-1 at 100 µg mL^−1^ showed incomplete separation of cells from each other, as indicated by the white arrows in Fig. 4 I C1–C3. Apoptotic cells with a fragmented nucleus are shown in Fig. 4 I C1–C2 and indicated by an orange arrow.

The cells after the treatment with fluconazole also showed incomplete separation resulting in the formation of chains, as indicated by the white arrows at both 10 µg mL^−1^ (Fig. 4 II B1–B3) and 20 µg mL^−1^ (Fig. 4 II C1–C3). The orange arrow in Fig. 4 II B2 indicates the nucleus of an apoptotic cell.

The presented images show the effect of disturbed cell division after the application of both Venetin-1 and fluconazole; however, disturbances in the separation of nuclear material during cell division were observed only after the Venetin-1 application. After the application of fluconazole, the cells formed chains, but no genetic material was observed in the intercellular constriction. These observations indicate a different mechanism of action of both preparations, despite the similar microscopic observations.

### Cell division analysis using Cryo-SEM and SEM

**Figure 5:**
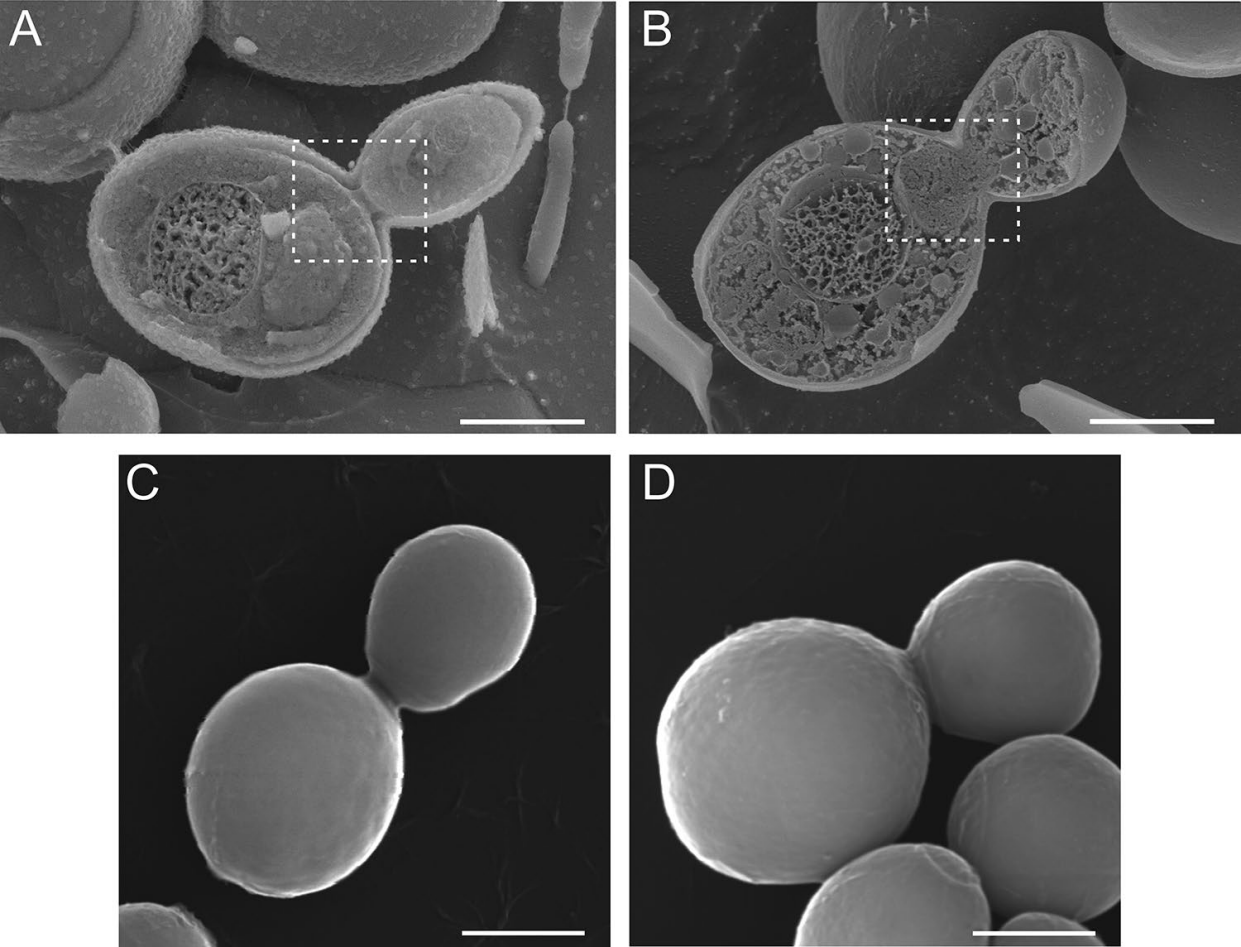
*C. albicans* cells imaged by Cryo-SEM (**A**, **B**) and SEM (**C**, **D**): (**A**)—cross-section of budding *C. albicans* cells from the control culture; (**B**)—cross-section of budding *C. albicans* cells after the incubation with Venetin-1 at 100 µg mL^−1^; (**C**)- cells of *C. albicans* from the control culture; (**D**)—cells after the incubation with Venetin-1 at 100 µg mL^−1^. The scale bar represents 2 µm.

*C. albicans* cells undergoing the budding process were also analyzed by the SEM and Cryo SEM microscopic techniques (Fig. 5). A cross section of the control culture cells is shown in image Fig. 5A. Proper budding is visible, in which the genetic material is fully separated between the parent and daughter cells (area marked with a square). The cells subjected to the incubation with Venetin-1 at 100 µg mL^−1^ are visualized in image on Fig. 5B. This photo shows incompletely separated genetic material between the parent cell and the daughter cell (marked with a white frame), which corresponds to the images shown in Fig. 4I. The SEM images of the control culture show normal cell division with a clearly visible division ring (Fig. 5C). Image on Fig. 5 D shows yeast cells after treatment with Venetin-1 at 100 µg mL^−1^ with incomplete cell separation. The SEM images are in agreement with the images obtained with the Cryo-SEM method.

### Analysis of *C. albicans* mitochondria

#### Microscopic analysis of mitochondria

**Figure 6:**
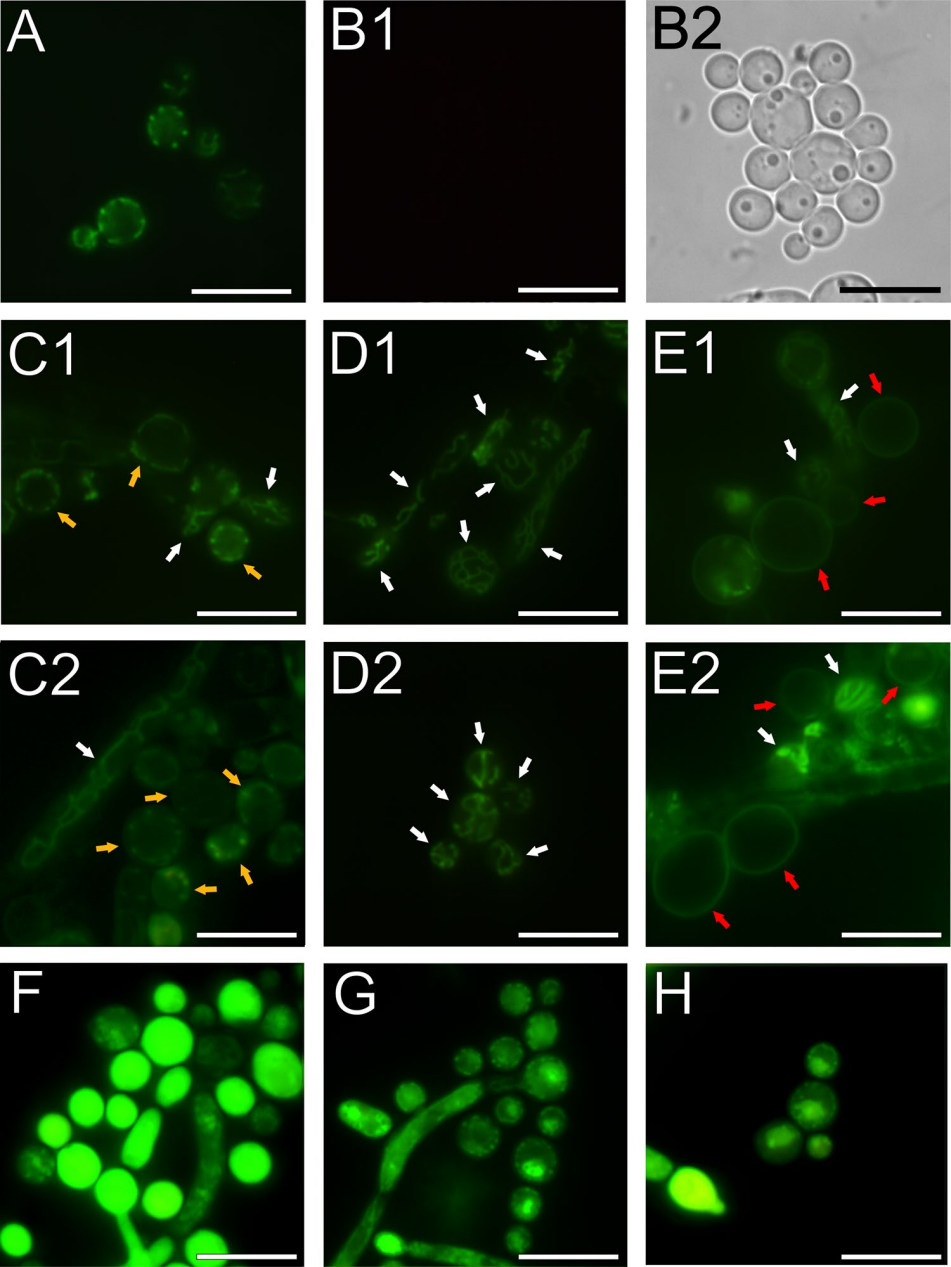
Rhodamine 123 staining of *C. albicans cells.* (**A**)—positive control cells with small round mitochondria; (**B1**)–(**B2**)—negative control cells treated with sodium azide. Picture (**B2**) is a visualization of cells in the transmitted light microscope, equivalent of picture (**B1**) from the fluorescence microscope. (**C1**)–(**C2**)—*C. albicans* cells treated with Venetin-1 at 25 µg mL^−1^, (**D1**)–(**D2**)—with Venetin-1 at 50 µg mL^−1^, (**E1**)–(**E2**)—with Venetin-1 at 100 µg mL^−1^; (**F**)–*C. albicans* cells treated with fluconazole at 5 µg mL^−1^, (**G**)—with fluconazole at 10 µg mL^−1^, (**H**)—with fluconazole at 20 µg mL^−1^. Yellow arrows show cells with a normal shape of mitochondria; white arrows indicate cells with elongated mitochondria; red arrows indicate cells that do not emit any inner fluorescence due to inactive mitochondria. The scale bar represents 10 µm.

The analysis performed using the Rhodamine 123 dye visualized the mitochondria of the control cells as small green points located on the internal peripheral of the cells (Fig. 6A). The cells of the negative control incubated with sodium azide did not emit observable fluorescence (Fig. 6B1). Image on Fig. 6 B2 is equivalent to image B1 from the transmitted light microscope. After the incubation with Venetin-1 at 25 µg mL^−1^ cells with elongated mitochondria (Fig. 6C1–C2, marked with white arrows) and cells with normal mitochondria (Fig. 6C1–C2, marked with yellow arrows) were observed. After applying Venetin-1 in an amount of 50 µg mL^−1^, mitochondria were often visualized as long, folded, green luminous structures filling the cell lumen (Fig. 6D1–D2). The culture subjected to the action of Venetin-1 at 100 µg mL^−1^ additionally exhibited cells with no inner fluorescence, which suggests that their mitochondria were inactive (Fig. 6E1–E2, marked with red arrows). Cells with elongated mitochondria are marked with white arrows in the same images. The incubation with fluconazole did not induce significant changes in the size or localization of mitochondria (Fig. 6F–H). Some cells exhibited intensive green fluorescence due to the high uptake of the fluorochrome, which was caused by the increased permeability of the cell membrane after the fluconazole treatment.

#### Analysis of mitochondria in *C. albicans* cells using flow cytometry

**Figure 7:**
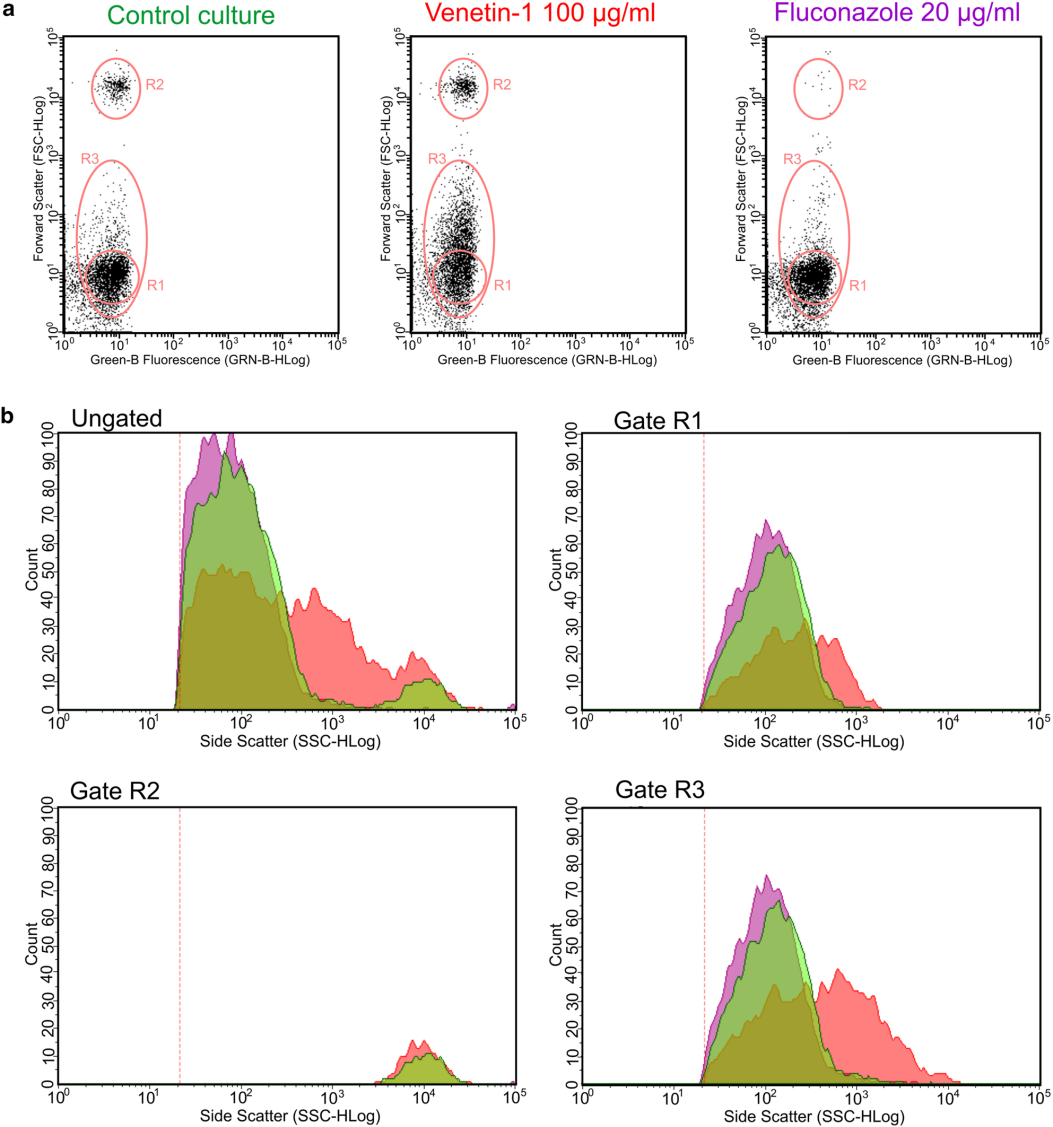
(**a**) Flow cytometry analysis of active mitochondria with Rhodamine 123. Dot-plots of the control culture, cells after the treatment with Venetin-1 at 100 µg mL^-1^, and in the variant with fluconazole at 20 µg mL^-1^. R1- cells with regular mitochondria; R2- aggregates; R3- cells with active mitochondria. (**b**) Gated regions after flow cytometry analysis: R1-cells with regular mitochondria; R2- aggregates; R3- cells with active mitochondria. Histograms present the profile of the cell cultures in the gated regions. Green—control sample, red—cells after the treatment with Venetin-1 at 100 µg mL^-1^, purple—cells subjected to fluconazole at 20 µg mL^−1^.

Flow cytometry was used to analyze active mitochondria with Rhodamine 123, and the results are presented in Fig. 7a. The upper dot plots show differences between the cultures in gated regions: R1—regular cells, R2—aggregates, and R3—cells with active mitochondria. In the R3 region, which is not contained in R1, the mitochondria of the cells are abnormal. The percentage of active cells (Gate R3) in all cultures was similar: 60.9% in the control culture, 64.34% after the incubation with Venetin-1 (at 100 µg mL^−1^), and 66.22% in the treatment with fluconazole (at 20 µg mL^−1^). A significant difference was noted in the number of regular cells after the treatment with Venetin-1 (36.1%) in comparison to the control culture cells (52.7%) and cells treated with fluconazole (57.64%). Changes in the R2 gate were also clearly visible, with values of 6.38% in the control culture, 8.42% in the cells treated with Venetin-1, and 0.22% in the culture incubated with fluconazole.

The side scatter relates to the complexity or granularity of the cell, including mitochondria. The histograms (Fig. 7b) present different profiles in the cell culture incubated with Venetin-1, which indicates that the structure of mitochondria changed after the treatment with the preparation. The observations did not show changes in mitochondrial morphology after the fluconazole treatment, in comparison to the control fungal culture.

### Phase contrast microscopy of vacuoles

**Figure 8:**
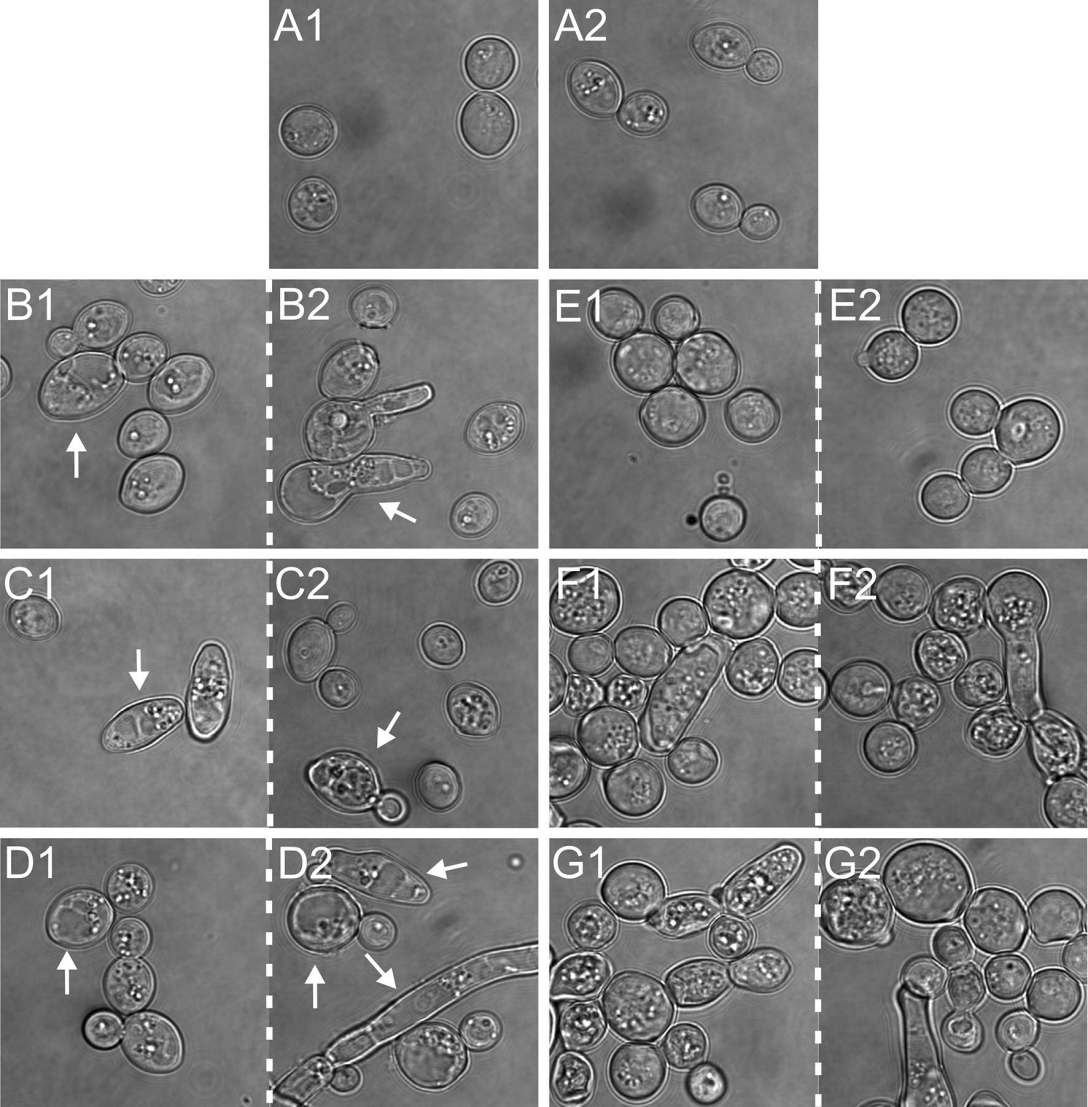
Phase contrast microscopy of *C. albicans* cells. (**A1**)–(**A2**)—*C. albicans* control cells; (**B1**)–(**B2**)—*C. albicans* after the incubation with Venetin-1 at 25 µg mL^−1^; (**C1**)–(**C2**)—with Venetin-1 at 50 µg mL^−1^; (**D1**)–(**D2**)—with Venetin-1 at 100 µg mL^−1^; (**E1**)–(**E2**)—*C. albicans* after the incubation with fluconazole at 5 µg mL^−1^; (**F1**)–(**F2**)—with fluconazole at 10 µg mL^−1^; (**G1**)–(**G2**)—with fluconazole at 20 µg mL^−1^. The arrows indicate enlarged vacuoles in *C. albicans* cells after the action of the Venetin-1 complex. The scale bar corresponds to 5 µm.

Phase contrast microscopy is a useful method for observation of objects that do not show natural contrast, including cell vacuoles. The microscopic image of the control *C. albicans* cells revealed cells with the correct oval shape (Fig. 8A1–A2). The cells exposed to the Venetin-1 complex at the concentrations of 25, 50, and 100 µg mL^−1^ were characterized by enlarged vacuoles observed in both blastoconidial and hyphal cells (Fig. 8B1–B2, C1–C2, D1–D2, changed vacuoles are indicated by arrows). Furthermore, altered vacuoles were more numerous and filled most of the treated cell (Fig. 8B2, D1–D2). In turn, fluconazole did not cause formation of enlarged vacuoles in the *C. albicans* cells at any of the concentrations used (5, 10, 20 µg mL^−1^), but there were cells with numerous granularities and a significantly enlarged size, relative to the control cells (Fig. 8E1–E2, F1–F2, G1–G2).

### Analysis of *C. albicans* cell viability using flow cytometry

**Figure 9:**
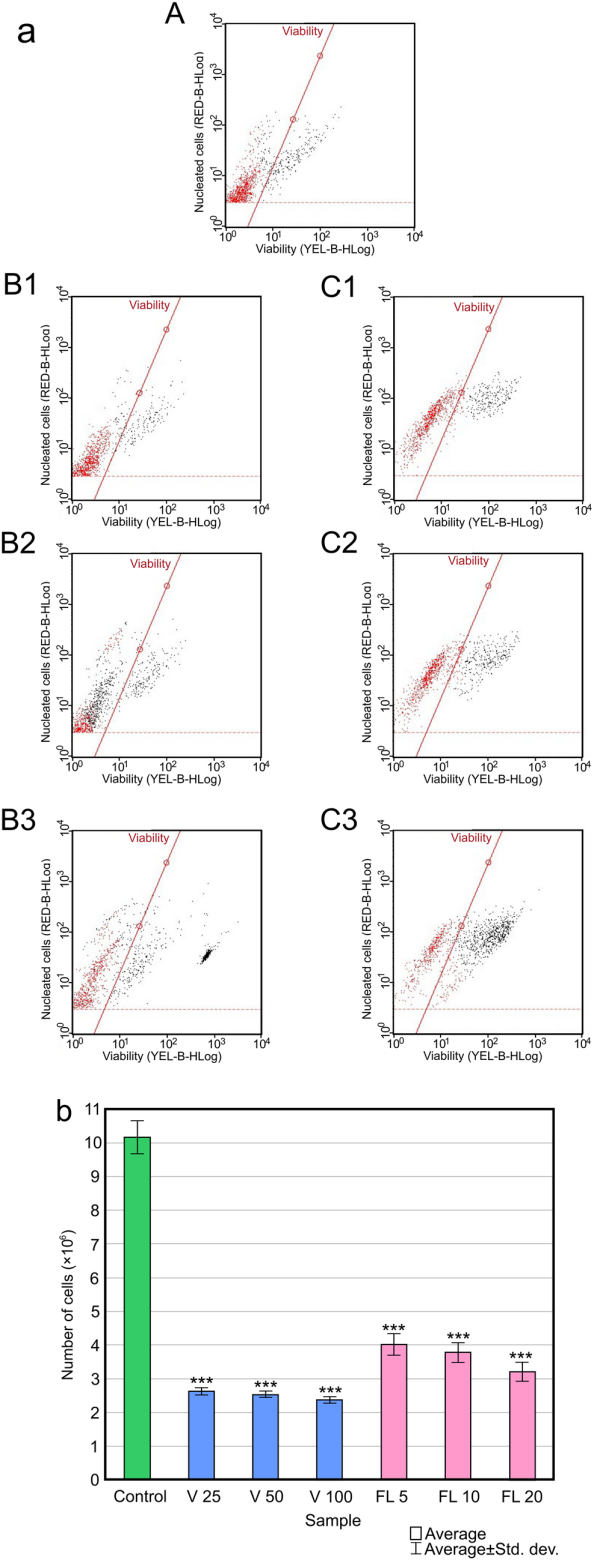
(**a**) Flow cytometry data of *C. albicans* cell cultures: (**A**)—control cell culture; (**B1**)—cell culture treated with Venetin-1 at 25 µg mL^−1^, (**B2**)—with Venetin-1 at 50 µg mL^−1^, (**B3**)—with Venetin-1 at 100 µg mL^−1^; (**C1**)—cell culture treated with fluconazole at 5 µg mL^−1^; (**C2**)—with fluconazole at 10 µg mL^−1^, (**C3**)—with fluconazole at 20 µg mL^−1^. The red color corresponds to viable cells and the black color indicates apoptotic cells. (**b**) total number of cells in the cultures. Control—control *C.* *albicans* cells; V25, V50, V100—*C. albicans* cells treated with Venetin-1 at 25, 50, 100 µg mL^-1^; FL5, FL10, FL20—cells treated with fluconazole at 5, 10, 20 µg mL^−1^; *** *P* < 0.01 (HSD Tukey Test).

The flow cytometry technique was used to analyze the viability of the cells treated with Venetin-1 and fluconazole (Fig. 9). The flow cytometry image of the control culture cells is shown in picture I A; the viability was estimated at 76.68%. Images on Fig. 9B1–B3 show data obtained from the analysis of the cell cultures incubated with Venetin-1 at the final concentrations of 25 µg mL^−1^ (Fig. 9a B1), 50 µg mL^−1^ (Fig. 9a B2), and 100 µg mL^−1^ (Fig. 9a B3). The percentage of apoptotic cells was 17.18% after the treatment with Venetin-1 at 25 µg mL^−1^ (Fig. 9a B1), 56.91% at 50 µg mL^−1^ (Fig. 9a B2), and 50.78% at 100 µg mL^−1^ (Fig. 9a B3). Image on Fig. 9a B3 shows a separate group of dead cells. Dot plots on Fig. 9a C1–C2, and C3 present data obtained after the analysis of the fluconazole-incubated cells at the concentrations of 5, 10, and 20 µg mL^−1^. The percentage of apoptotic cells increased with the increasing fluconazole concentration and amounted to 22.11% in the cell culture incubated with fluconazole at 5 µg mL^−1^, 26.88% at 10 µg mL^−1^, and 58.41% at 20 µg mL^−1^. Figure 9b shows the total number of control cells and in the cultures subjected to the different concentrations of Venetin-1 and fluconazole. There was a significant difference between the treated and untreated cells.

### FTIR spectroscopy analysis

**Figure 10:**
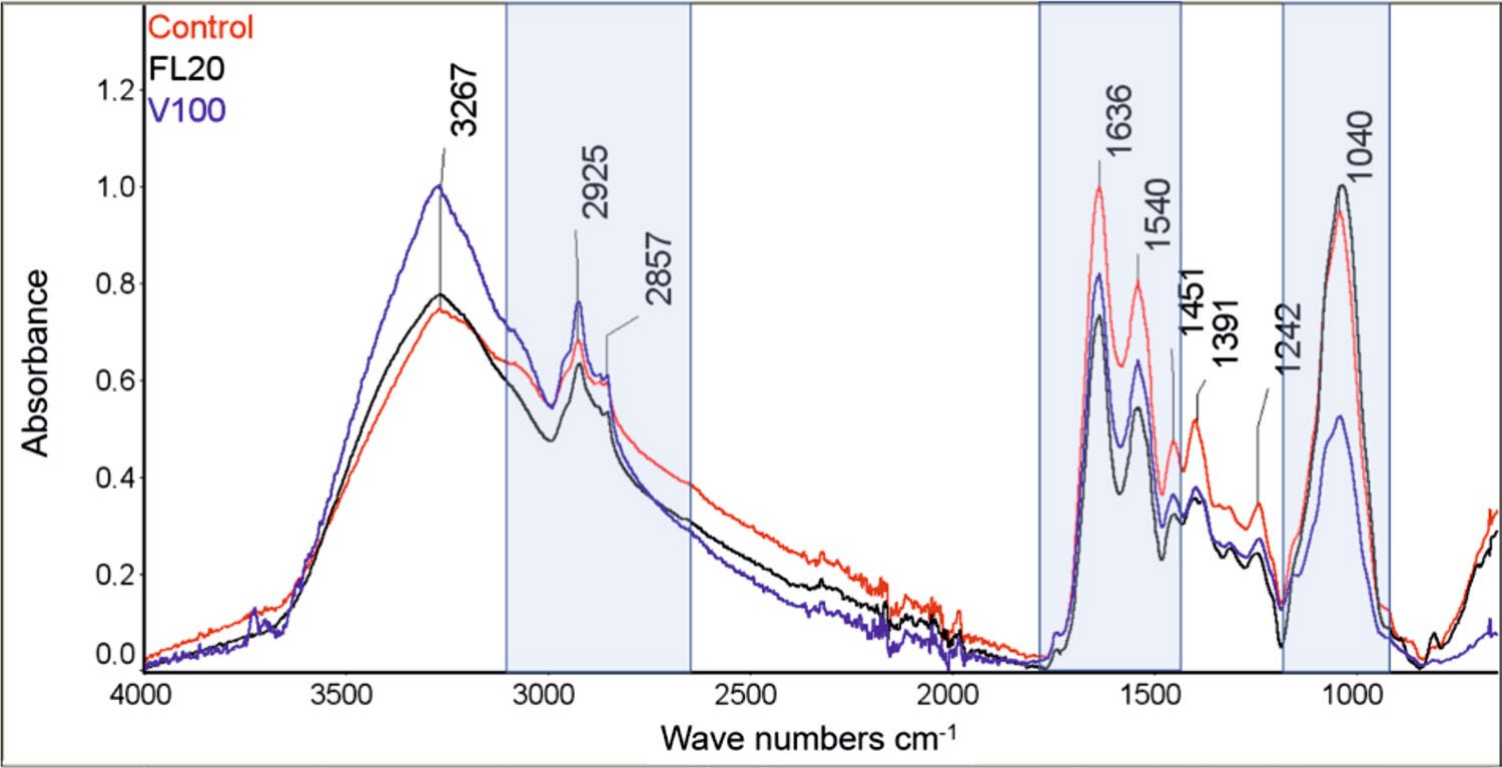
FTIR spectrum for the *C. albicans* control culture (Control) and after the treatment with Venetin-1 at 100 µg mL^−1^ (V100) and fluconazole at 20 µg mL^−1^ (FL20).

Three spectral regions were analyzed in the FTIR spectrum: in the range of 3200–2800 cm^−1^, 1720–1400 cm^−1^, and 1200–950 cm^−1^ (Fig. 10). Signals characteristic for vibrations corresponding to bonds occurring in lipids were observed in the range of 3200–2800 cm^−1^ in the FTIR spectrum. The range of 1720–1400 cm^−1^ exhibited peaks corresponding to the vibrations characteristic for bonds occurring in proteins, while bands characteristic of polysaccharides were observed in the range of 1200–950 cm^−1^. The tests showed an increase in signals in the region of 2800–3500 cm^−1^ after the treatment of the *C. albicans* sample with Venetin-1 at 100 µg mL^−1^ (Fig. 10). A significant drop in signals at 900–1800 cm^−1^ was observed as well. In the case of the treatment of the *C. albicans* sample with fluconazole 20 µg mL^−1^, a decrease in the signal intensity only in the range of 1800–1200 cm^−1^ was observed. Based on the literature data, selected signals were analyzed with the assignment of characteristic chemical bonds. The results are presented in Table 2.

**Table. 2.** Analysis of selected signals with assignment of characteristic chemical bonds^44^.

Wavenumber (cm^−1^)	Peak position (cm^−1^)	Bonds
3200–2800	2925	CH_3_ from lipids
	2857	CH_2_ from lipids
1720–1400	1636	Amide I
	1540	Amide II
1200–950	1040	Glycogen

## Discussion

It was revealed in earlier studies that the complex obtained from *D. veneta* coelomic fluid acts on the *C. albicans* cell wall and membrane. Its effect was compared to that of fluconazole, whose action is well known. Fluconazole is an azole antifungal drug from the triazole derivative group. The mechanism of action of drugs that belong to this group of synthetic compounds is based on the inhibition of the enzyme responsible for the biosynthesis of ergosterol, which is one of the components that build the fungal cell membrane^10,45^. This metabolic disturbance increases the permeability of the cell membrane. The cell membrane loses its protective functions and the cell stops growing, which results in the death of the fungus.

The current medicines and antibiotics are becoming less effective against pathogens. This is related the fact that fungi are developing antibiotic resistance against commonly used drugs such as azoles^46^. Fluconazole is a first-choice medicine in antifungal treatment. Prolonged intake or numerous treatments with this antibiotic may lead to *Candida* resistance^47–49^. Fluconazole is used for prophylaxis of mycoses in immunocompromised patients, including patients in intensive care units, organ transplant patients, and those undergoing chemotherapy and radiotherapy. It has been reported that clinical *C. albicans* isolates are resistant to fluconazole in approx. 30–40%^50,51^. It has been shown that there is an urgent need for development of new antifungal preparations that can be administered to patients with *C. albicans* infection.

The natural environment has always been a source of inspiration in search of substances with beneficial properties. They can be found in all kingdoms of life: bacteria and fungi produce antibiotics, while essential oils and a variety of favorable molecules can be extracted from plants^45^. Animals also produce substances that have been improved during their evolution to protect them from diseases. Earthworms, which are among the longest living animals on Earth, are such organisms.

In our previous studies^34^, we described *C. albicans* cells stained with the Calcofluor white fluorochrome after the action of the protein-polysaccharide fraction of earthworm coelomic fluid. It was observed that the cell wall stained unevenly, indicating a different thickness of the chitin layer in the cell wall of yeast cells treated with the active agent. In the present study, the formation of different cell aggregates was microscopically imaged as well. The type and quantity of multicellular aggregates were analyzed both after the incubation with the complex and after the incubation with fluconazole. These analyses showed differences in the number of multicellular formations, which indicate a different mechanism of action against the *C. albicans* cell wall. This was also confirmed by the other analyses.

The Congo red fluorochrome was used simultaneously in the analyses. It can form a complex with (1,3)-β-glucan, which makes the surface of fungal cell walls to fluoresce red under a fluorescence microscope. The Venetin-1 complex caused exposure of (1,3)-β-glucan, while fluconazole did not expose this cell wall component. Slight fluorescence was observed only in the division scars. It could be related to the phenomenon described by Pfaller and Riley^52^ that *C. albicans* cells treated with fluconazole contain significantly less glucan in their walls. Much research has investigated the synergistic action of various compounds with fluconazole to expose the β-glucan layer^47,53–55^. In our case, this exposure was observed only after the action of the complex, which was confirmed by Cryo-SEM. The flattening of the outermost layer of the Venetin-1 treated cells may indicate destruction of the mannoprotein layer beneath which the layer of β-glucans is located, which explains the red fluorescence observed after using the Congo red fluorochrome. Recognition of the β-glucan component of the fungal cell wall is a key element of the host immune system; therefore, compounds that expose glucan may become valuable drugs^53,56^.

The FTIR spectroscopic tests of *C. albicans* performed after the application of Venetin-1 showed a decrease in signal intensity in the range of 1720–1400 cm^−1^ and 1200–950 cm^−1^. These bands correspond to vibrations characteristic of chemical bonds found in proteins and polysaccharides, which are components of the *C. albicans* cell wall. The decrease in the intensity of peaks characteristic of proteins may indicate destruction of the mannoprotein layer of the *C.* *albicans* cell wall as a result of the action of Venetin-1. A decrease in the intensity of signals characteristic of polysaccharides, which are also a component of the *C. albicans* cell wall, was also observed. The Cryo-SEM images showed such effect of cell wall destruction as well. The FTIR spectroscopic studies also showed an increase in the intensity of signals corresponding to the vibrations characteristic of bonds occurring in lipids. This confirms the hypothesis regarding the destruction of the mannoprotein layer and the β-glucan layer of the *C. albicans* cell wall and the exposure of the lipid layer of the cell membrane.

The effect of the complex on the cell wall and membrane of *C. albicans* cells can be seen in the analysis of the fungal cells using phase contrast microscopy, where cells with unnaturally enlarged vacuoles were observed, suggesting changes in membrane permeability. No such effect was observed after the incubation of the fungal cells with fluconazole, which indicates a different mechanism of action of this antibiotic. Taking into account our earlier studies, which showed that lysenin and lysenin-related protein 2 are the main proteins building the complex^39^, the theory about changes in the cell membrane permeability is understandable. It is known that lysenin proteins are mainly involved in defense against both eukaryotic and prokaryotic pathogens. Lysenin binds to the membrane receptor sphingomyelin. Upon attachment to the membrane after conformational change, the oligomer is incorporated into the membrane^57^.

Cell division disorders were also analyzed using a mixture of Hoechst and Propidium iodide, which can label the genetic material and show apoptotic and necrotic cells. During the correct division in the stem cell, the mitotic division of the nucleus into two cells takes place. One nucleus, together with part of the cytoplasm, diffuses into the resulting bulge called the bud. The bud gradually grows and is separated from the stem cell by the cell wall. In the cells treated with the coelomic fluid complex, undistributed genetic material was often observed between the mother and daughter cells. The daughter cells grew undivided from the parent cell, and the nuclear material was visible in the constriction between the cells. This effect was not observed in the case of incubation with fluconazole. The sections of fungal cells treated with Venetin-1 and imaged by Cryo-SEM and SEM showed incomplete cell separation during budding, compared to control cells, where the cell wall and membrane clearly separated the cells during division. After the action of the complex, the daughter cell grew to a large size, unable to detach from the parent cell, and as a result, cell doublets with a constriction between the cells were visible. This indicates an abnormal division of nuclear material with a simultaneous disturbance of the wall and membrane system. The cell grew with a deficit of components needed to complete the division of the daughter cell. *C. albicans* cells treated with fluconazole underwent division with complete separation of the nuclear material between the cells, which is confirmed by the literature data^34^.

The data obtained after the Rhodamine 123 staining showed that the Venetin-1-treated *C. albicans* cells had clearly visible changes in the mitochondrion shape and were characterized by loss of mitochondrial membrane potential. Research conducted by Fiołka et al.^39^ demonstrated that fungal cells treated with the complex had elevated levels of Reactive Oxygen Species (ROS) and a higher level of proteins involved in ROS degradation. Oxidative stress may result in damage to DNA, proteins, and lipid compounds of the cell structures^58^. An increased ROS level in the mitochondrion leads to opening ion channels of the mitochondrial membrane and, in consequence, to a phenomenon called RIRR (ROS-induced ROS release), where disruption of the mitochondrial membrane potential causes higher production of ROS in the electron transport chain^59,60^. Oxidative stress may result in apoptosis or necrosis of cells, and both ways of cell death after the treatment of *C. albicans* with the Ventin-1 (previously described as coelomic fluid fraction) were observed^34,39,60^. The fluconazole-treated *C.* *albicans* cells did not exhibit any changes in the mitochondrion morphology in Rhodamine 123 staining. The effect on mitochondria exerted by oxidative stress induced by fluconazole treatment is based on impairment of their function by suppressing mitochondrial electron transport, which is reversible^61–64^. Temporal loss of mitochondrial function is described in literature as the mechanism of fluconazole resistance^62,65^.

Both the Venetin-1 complex and fluconazole induced the formation of multicellular aggregates; however, flow cytometry revealed differences between the cell cultures. The analysis of *C.* *albicans* viability was performed only in cells that did not form large agglomerates or complex multicellular structures, because the design of the cytometer allows analyses of single cells. However, in the case of the action of the complex and the action of fluconazole, the aggregate formation effect is similar, and the flow cytometry results can be compared with each other, because identical settings of the analysis were applied in every experiment.

The analysis of active mitochondria conducted with flow cytometry showed differences between cell cultures treated with Venetin-1 and fluconazole. Despite the similar levels of cells with active mitochondria, the treatment with the coelomic fluid complex lowered the percentage of regular cells. This indicates that Venetin-1 caused alterations in the mitochondria, while fluconazole did not. This suggests that the mechanism of action of Venetin-1 differs from that of fluconazole. The cytometry results support the observations of the Rhodamine 123-stained cells examined by fluorescence microscopy, in particular the elongation of mitochondria in the cultures treated with Venetin-1 and no visible effect on mitochondria after the incubation with fluconazole.

The cell viability analysis carried out using flow cytometry showed a similar level of apoptotic cells in the cultures incubated with Venetin-1 at 100 µg mL^−1^ and fluconazole at 10 µg mL^−1^. The data obtained in this experiment also showed that, the highest concentration of Venetin-1 caused necrotic cell death, which was not observed after the fluconazole treatment. Both preparations significantly lowered the number of cells, in comparison to the control culture.

In the search for new therapeutics for use in the treatment of infectious diseases, the key issue is the lack of cytotoxicity that would limit their applicability. In the case of fluconazole, there are reports of its hepatotoxicity and neurotoxicity^66,67^ as well as cytotoxic and genotoxic effects on the monkey kidney cell line^68^ and cultured human peripheral blood mononuclear cells^69^. In turn, our previous research indicates that heat-treated *D. veneta* coelomic fluid and Venetin-1 do not have cytotoxic effects against human fibroblasts^70^, normal human colon epithelial cells (CCD 841 CoTr)^36^, human platelet-rich plasma^37^, and human bronchial epithelial cells (BEAS-2B)^35^. When searching for new therapeutics for use in medicine, one should take into account not only the effective action and the lack of cytotoxicity, which saves normal cells, but also the costs and efficiency of the method for isolation of the active compound. Taking into account these requirements, the Venetin-1 complex obtained from the coelomic fluid ideally meets the requirements for a candidate for biomedical research. In further analyses, we intend to explore the effects of the complex on various *Candida* species, explore the structure of the complex, and analyze its action in a living organism in a mouse model.

Summing up, the analyzed earthworm preparation with the different mechanism of action than that of a typical antibiotic prompts further investigations thereof, as it seems to be a promising antifungal compound.

## Data Availability

The raw data used and analyzed during the current study available from the corresponding author on reasonable request.
